# High uptake of COVID-19 vaccines among healthcare workers in urban Uganda

**DOI:** 10.1371/journal.pone.0277072

**Published:** 2024-04-16

**Authors:** Nasimu Kyakuwa, Geofrey Kimbugwe, Flavia Nakanjako, Hamza Kalute, Simon Mpooya, Christine Atuhairwe, Laurent Perez, Bernard Kikaire

**Affiliations:** 1 Uganda Virus Research Institute, Entebbe, Uganda; 2 MRC/UVRI & LSHTM Uganda Research Unit, Entebbe, Uganda; 3 Uganda Martyrs University, Entebbe, Uganda; 4 University of Lausanne, Lausanne, Switzerland; 5 Makerere University College of Health Sciences, Kampala, Uganda; Jiangsu University, CHINA

## Abstract

**Objectives:**

The aim of the study was to describe the facilitators, barriers to and level of uptake of COVID-19 vaccines among healthcare workers in primary healthcare facilities in an urban setting in Uganda.

**Materials and methods:**

We conducted a cross-sectional study among HCWs in private and public health facilities in Entebbe municipality between July 2021 and August 2021. Data was collected using a structured questionnaire that was shared, via an online link, to consented participants. Uptake of the vaccines among healthcare workers was analysed as proportions, and logistic regression was used to analyse barriers and facilitators to uptake of COVID-19 vaccines.

**Results:**

The study enrolled 360 participants, with 61.7% (n = 222) females. A total of 236 (65.6%) healthcare workers had received at least one dose of COVID-19 vaccine, with higher uptake among females 64% (n = 151). Age above 40 years (OR 2.16), working in a government healthcare facility (OR 3.12), participating in COVID-19 vaccine related activities (OR 4.62), and having tested for SARS-COV-2 (OR 3.05) increased the odds of having been vaccinated. Working in small roadside clinics reduced the odds of being vaccinated by almost 70%, while HCWs in government health services were 3.1 times more likely to have been vaccinated. History of having cared for a COVID-19 patient and having a positive SARS-COV-2 test result did not influence the uptake of the vaccines in the study population.

**Conclusion:**

Vaccine uptake among HCWs was close to the World Health Organisation (WHO) recommended uptake of 70% by mid-2022.

## Introduction

Vaccination is a highly cost-effective strategy for controlling infectious diseases in populations [1], and has led to a significant reduction in the incidence of childhood vaccine preventable diseases such as measles, polio, and pertussis [1, 2], as well as other infectious diseases like influenza [3, 4]. In addition to providing the direct effect of protection to the vaccinated individual, high vaccination coverage rates provide indirect benefits to the community through herd immunity [5, 6], hence decreasing the risk of infection among susceptible, un-vaccinated individuals within the community [7]. Despite the effectiveness of vaccination as a public health intervention, [8, 9] vaccine hesitancy persists in both the general population and among health care workers (HCWs) [10], with some individuals perceiving vaccines as unsafe and unnecessary [11]. This hesitancy is particularly prevalent among those with higher levels of education [12] and is highly context-, vaccine-, and profession specific [13]. Vaccine hesitancy among HCWs remains a public health threat [14] as unvaccinated HCWs are at a risk of contracting infections from their patients, and patients could similarly contract infections from HCWs. Globally, vaccination against SARS-COV-2 met hesitancy or low uptake which threatens the attainment of the World Health Organization (WHO) recommended uptake of 70% by mid-2022. This hesitancy is particularly pronounced among those who perceive themselves to be at low risk of severe corona virus disease. Rates of COVID-19 vaccine acceptance vary from region to region with Africa having the lowest rates of COVID-19 vaccine acceptance [15]. A systematic review of COVID-19 vaccine acceptance among HCWs worldwide showed a wide range of vaccine acceptance rates, with the highest rates (78.1%) in Israel and the lowest (27.7%) in the Democratic Republic of the Congo [15] with many countries falling in between. A study carried out among HCWs in Los Angeles showed that acceptance of COVID-19 vaccine varied with the role of HCWs with physicians and research scientists being more likely to take vaccines than others [16]. While several studies have explored intentions to be vaccinated in the general population, few studies have assessed actual vaccine uptake, particularly among HCWs. A study by Juliet *et al*. 2022 [17] reported vaccine uptake of 65.3%, however, this study was among eye health care workers at a tertiary hospital. No studies have reported uptake of vaccines among the general health care workers at peripheral facilities. Therefore, this study aimed to assess the barriers and facilitators to the uptake of COVID-19 vaccines among HCWs within an urban setting in Entebbe municipality in Uganda.

## Materials and methods

### Study design

This was a cross-section study conducted between July and August 2021 in both the private and government primary health care health facilities and the research center within Entebbe Municipality, Wakiso District in central Uganda. The regional referral hospital was excluded due to a similar study that was ongoing at that time.

### Study setting

Entebbe Municipality is an urban center in Wakiso district in central Uganda. It has a population of 67271 people. The municipality has one research centre, one regional referral hospital and about 40 health facilities of which the majority (about 33) are privately owned including Private Not for Profit (PNFP) and Private for Profit (PFP).

### Study participants

Participants were HCWs in primary healthcare facilities and were categorized as medical or non-medical HCWs. The medical HCWs included medical doctors, nurses, nursing assistants, paramedics, social workers, and research scientists, while the non-medical HCWs included health centre managers, receptionists, cleaners, porters, and janitors. All HCWs found at the facility at the time of data collection were eligible to participate in the study. Sample size was calculated using Kish Lesley formula for a single proportion, with vaccine hesitancy rate of 72.3% [15], a precision of 5%, and a non-response correction of 20% since study was conducted during the COVID-19 lock down period. This led to a total sample size of 364 including the 20% non-response.

### Data collection

Data was collected between 1^st^ July to 03^rd^ August 2021. A structured questionnaire developed using Research Electronic Data Capture (REDCap) was used to collect data. The questionnaire was adapted from the WHO Strategic Advisory Group on Experts (SAGE) on Immunization survey tool [18]. HCWs that agreed to participate and provided consent had the questionnaire link shared through email or WhatsApp. Participants that had no computer or smart phone were offered the study’s smart phone to complete the survey. Study participants were selected by convenient sampling as there were no established healthcare worker’s contact lists within the municipality. The study was also conducted during the national lock down therefore only participants who were on duty were approached.

### Statistical analysis

Data was analyzed using STATA 12. Uptake of COVID-19 vaccines was calculated as a proportion of HCWs that had received the vaccine compared to total number of participants. Vaccine uptake was described as having received at least one dose of any COVID-19 vaccines available in the country at the time of the study. Chi square test was used at bivariate analysis and variables that had a p-value ≤ 0.2 were considered for multivariate analysis using logistic regression.

### Ethical consideration

Ethical approval was obtained from the Uganda Virus Research Institute’s Research and Ethics Committee (UVRI-REC) approval number GC/127/845. Informed written consent was sought and obtained from all study participants.

## Results

### Socio-demographic characteristics of respondents

The study enrolled 360 participants of whom 222 (61.7%) were female and had an average age of 31.0 years (SD 7.95). Majority (79.7%) had attained either a degree and or masters, and about 69% were medical health care workers. The HCWs were mainly from private health care facilities (63.9%). Table 1 shows the details of the social demographic characteristics of the respondents.

**Table 1:** Socio-demographic characteristics and COVID-19 vaccine uptake.

Category	Vaccine Uptake		
	No (%)	Yes (%)	Total (%)
**Gender**			
Male	53 (42.7)	85 (36.0)	138 (38.3)
Female	71 (57.3)	151 (64.0)	222 (61.7)
**Age group**			
18–29 Years	83 (66.9)	108 (45.8)	191 (53.1)
30–39 Years	31 (25.0)	80 (33.9)	111 (30.8)
≥40 years	10 (8.1)	48 (20.3)	58 (16.1)
**Level of education**			
Certificate	29 (23.4)	44 (18.6)	73 (20.3)
Bachelors/masters	95 (76.6)	192 (81.4)	287 (79.7)
**Job category**			
Medical	81 (65.3)	167 (70.8)	248 (68.9)
Non-medical	43 (34.7)	69 (29.2)	112 (31.1)
**Contacts(n = 248)**			
Primary contacts**	43 (53.1)	108 (64.7)	151 (60.9)
Secondary contacts***	38 (46.9)	59 (35.3)	97 (39.1)
**Level of service of the health facility**			
Hospitals	17 (13.7)	40 (16.9)	57 (15.8)
Health centre III & IV	30 (24.2)	114 (48.3)	144 (40.0)
Small Roadside clinics	77 (62.1)	82 (34.7)	159 (44.2)
**Type of ownership**			
Private not for profit (PNFP)	39 (31.5)	45 (19.1)	84 (23.3)
Private for profit (PFP)	72 (58.1)	74 (31.4)	146 (40.6)
Government	13 (10.5)	117 (49.6)	130 (36.1)
**Had ever cared for COVID-19 patients**			
No	70 (56.5)	156 (66.1)	226 (62.8)
Yes	54 (43.5)	80 (33.9)	134 (37.2)
**Had ever tested for SARS-COV-2**			
No	58 (46.8)	31 (13.1)	89 (24.7)
Yes	66 (53.2)	205 (86.9)	271 (75.3)
**SARS-COV-2 test results**			
Negative	53 (80.3)	177(86.3)	230 (84.9)
Positive	13 (9.7)	28 (13.7)	41 (15.1)
**Taken part in COVID-19 vaccine activities**			
No	91 (73.4)	59 (25.0)	150 (41.7)
Yes	33 (26.6)	177 (75.0)	210 (58.3)

** HCWs who interface with patients first,

***HCWs who interface with patients who have been screened or deal with biological materials obtained from patients

### COVID-19 vaccines uptake

Approximately two thirds, 65.6% (n = 236) of the respondents, had received at least one dose of COVID-19 vaccine. At bivariate analysis, respondents who were at least 40 years were more likely to have received the COVID-19 vaccines (P < 0.001). Other socio-demographic characteristics such as gender, level of education, job category, cadre and being a non-medical worker were not predictive of uptake of COVID-19 vaccines. The type of service provider, type of ownership, and area of operation at the health facility were important factors associated with uptake of COVID-19 vaccines in this study. Health workers in small roadside clinics were less likely to have been vaccinated than in other establishments (P = 0.016), while working in a government health facility (P < .001) and previous testing for the coronavirus (P <0.001) increased the odds of having been vaccinated. Details are depicted in Table 2.

**Table 2:** Determinants of COVID-19 vaccine uptake among health workers.

Category	Vaccine Uptake			OR (95% CI)	p-value	Adjusted OR (95% CI)	Adjusted p-value
	No (%)	Yes (%)	Total (%)				
**Gender**							
Male	53 (42.7)	85 (36.0)	138 (38.3)	1			
Female	71 (57.3)	151 (64.0)	222 (61.7)	0.75 (0.48–1.17)	0.213	0.85 (0.40–1.82)	0.691
**Age group**							
18–29 Years	83 (66.9)	108 (45.8)	191 (53.1)	1.			
30–39 Years	31 (25.0)	80 (33.9)	111 (30.8)	1.98 (1.19–3.28)	0.008	1.89 (0.82–4.34)	0.131
≥40 years	10 (8.1)	48 (20.3)	58 (16.1)	3.68 (1.76–7.72)	0.001	2.16 (0.75–6.18)	0.148
**Level of education**							
Certificate	29 (23.4)	44 (18.6)	73 (20.3)	1			
Bachelors/masters	95 (76.6)	192 (81.4)	287 (79.7)	1.33 (0.78–2.26)	0.288	0.98 (0.34–2.63)	0.343
**Job category**							
Medical	81 (65.3)	167 (70.8)	248 (68.9)	0.77 (0.48–1.23)	.290		
Non-medical	43 (34.7)	69 (29.2)	112 (31.1)	1			
**Contacts(n = 248)**							
Primary contacts**	43 (53.1)	108 (64.7)	151 (60.9)	1			
Secondary contacts***	38 (46.9)	59 (35.3)	97 (39.1)	0.61 (0.36–1.06)	0.081	0.66 (0.32–1.33)	0.248
**Level of service of the health facility**							
Hospitals	17 (13.7)	40 (16.9)	57 (15.8)	1			
Health centre III & IV	30 (24.2)	114 (48.3)	144 (40.0)	1.61 (.80–3.23)	0.177	0.52 (0.15–1.87)	
Small Roadside clinics	77 (62.1)	82 (34.7)	159 (44.2)	0.45 (0.23–0.86)	0.016	0.36 (0.13–1.00)	0.052
**Type of ownership**							
Private not for profit (PNFP)	39 (31.5)	45 (19.1)	84 (23.3)	1			
Private for profit (PFP)	72 (58.1)	74 (31.4)	146 (40.6)	0.89 (0.52–1.52)	0.673	0.82 (0.34–1.98)	
Government	13 (10.5)	117 (49.6)	130 (36.1)	7.8 (3.81–15.95)	<0.001	3.12 (1.06–9.17)	0.038
**Had ever cared for COVID-19 patients**							
No	70 (56.5)	156 (66.1)	226 (62.8)	1			
Yes	54 (43.5)	80 (33.9)	134 (37.2)	0.66 (0.42–1.030	0.073	0.68 (0.32–1.43)	
**Had ever tested for SARS-COV-2**							
No	58 (46.8)	31 (13.1)	89 (24.7)	1			
Yes	66 (53.2)	205 (86.9)	271 (75.3)	5.81 (3.46–9.74)	<0.001	3.05 (1.43–6.52)	0.004
**SARS-COV-2 test results**							
Negative	53 (80.3)	177(86.3)	230 (84.9)	1			
Positive	13 (9.7)	28 (13.7)	41 (15.1)	0.64 (0.31–1.33)	0.236		
**Taken part in COVID-19 vaccine activities**							
No	91 (73.4)	59 (25.0)	150 (41.7)	1			
Yes	33 (26.6)	177 (75.0)	210 (58.3)	8.27 (5.04–13.56)	<0.001	4.62 (2.34–9.08)	<0.01

** HCWs who interface with patients first,

***HCWs who interface with patients who have been screened or deal with biological materials obtained from patients

### Determinants of COVID-19 vaccines uptake in Entebbe Municipality

At multivariate analysis, being female reduced the odds of having been vaccinated by 15% (OR 0.85, 95% CI 0.40–1.82, p-value 0.691). Increasing age increased the odds of vaccination with participants 30–39 years being 80% (OR 1.89, 95% CI: 0.82–4.34, P value 0.131) and those above 40 years being more than two times (OR 2.16, 95% CI 0.75–6.18, P = 0.148) more likely to have been vaccinated. Working in small roadside clinics reduced the odds of being vaccinated by almost 70%, while HCWs in government health services were 3.1 times more likely to have been vaccinated (OR 3.12; 95% CI: 1.06–9.17, P = 0.038). History of being tested for corona virus infection increased the odds of having received the COVID-19 vaccine by 3 times (OR 3.05; 95% CI: 1.43–6.52, P = 0.004) and participating in the coronavirus vaccination activities increased the odds by more than 4 times (OR 4.62; 95% CI: 2.34–9.08, P value <0.01). Details are depicted in Table 2 below.

## Discussion

In this study, the uptake of COVID-19 vaccines among HCWs within Entebbe municipality was 65.6%. This uptake is close to the WHO globally recommended 70% vaccine uptake level by mid-2022 and similar to the findings of Juliet *et al*. 2022 who reported an uptake of 65.3% among eye healthcare workers [17]. This similarity in the uptake could be because both studies involved frontline healthcare workers whose risk perceptions may be similar. Our study reported a slightly lower uptake in comparison to Lubega *et al*. 2021 who reported an uptake of up to 70% among health care workers in two hospitals in Uganda [19]. Whereas Lubega studied health care workers in tertiary hospitals, this study was conducted among health care workers in primary health care units thus the differences in uptake of the vaccines. Our findings differ from a study in Nigeria that reported uptake rate of 33% that completed two doses of the COVID -19 vaccine [20]. The Nigeria study was conducted in the early days of COVID-19 vaccine introduction, when many unknowns about the vaccine existed, while our study was carried out much later and after the highly fatal increase in mortality due to the delta variant of the COVID-19 pandemic. It is probable that the relatively high uptake of vaccines by HCWs observed in our study was driven by the fear resulting from the ‘delta wave’. A multi-ethnic study in the UK healthcare workforce by Christopher *et al*. 2021 reported uptake rates of 64.5% [21], and this is similar to the findings of our study. A study by Maria *et al*. 2021 among emergency HCWs in US [22] reported vaccine uptake of 79% which is much higher than what we found. This could be due to differences in risk perceptions by the HCWs in the two studies explained by the Health Belief Model. Unlike in our study where the mean age was 31 years, the mean age of study participants in Maria’s was 41 years. It is well known that the risk of severe disease increases with increased age, therefore the difference in age could have led to a higher uptake in Maria’s study compared to ours. Furthermore, 29% of the participants had underlying health conditions, a variable that was not assessed for in this study.

Most studies both in Africa and globally have explored vaccine acceptance among HCWs and reported acceptance rates ranging between 50–70% [23–26]. In Pakistan, vaccine acceptance was reported at 70.2% [27] while in Canada, Stefania *et al*. 2021 reported vaccine acceptance rate of 80.9% [28], and China reported vaccine acceptance of 86.2% [29]. While these studies showed that health care workers were likely to take up COVID-19 vaccines once available, it should be noted that the actual uptake may be different from the intention [30, 31]. Studies in the United Kingdom of Saudi Arabia showed that the intention of HCWs taking vaccination was 70%, but when they studied actual uptake, the acceptance rate was 33.2% [26, 32]. However as noted above few studies have explored the actual vaccine uptake and this requires further investigation using follow up studies.

We found that being at least forty years increased the odds of taking up the COVID-19 vaccines. This is not surprising since the disease is known to preferentially affect the older people and other studies have reported similar findings [33–36]. This age related perception of risk also explains why younger health care workers were less likely to be vaccinated, since the risk of severe disease has been reported to be low among younger age groups [37] although some studies have reported severe diseases among younger age groups. Andrea *et al*. 2021 reported that almost 70% of the young ‘low risk’ with ongoing symptoms of COVID-19 had impairment in one or more organs four months after initial symptoms of SARS-CoV-2 infection [38]. This study seems to suggest that even though low risk individuals have lower mortality rates, their severity of disease is seen by organ temporary impairment and follow up of this category is of public health importance.

The findings of our study showed that the men were more likely to be vaccinated as compared to women, which agrees with other studies [22, 25]. Just like the age related risk perception, the male gender has been reported to suffer more severe acute respiratory distress syndrome compared to females [39–41] and this could have driven more men to seek vaccination.

Although a study in Western Uganda reported that having attained tertiary level of education increased the intention to be vaccinated [25] and Megan *et al*., 2021 in California reported that vaccine uptake was associated with higher level of education [42], we found that level of education did not affect uptake of vaccines among health care workers in Entebbe. Level of education may increase perception of risk, and since HCWs have a good understanding of risk, their education level may thus not be a factor affecting actual uptake of vaccination.

The convenience/reliability of vaccination services was critical in vaccine uptake. In this study, we found out that being a government worker was associated with increased uptake of vaccines than working in private for profit and private not for profit healthy facilities. Rollout of COVID-19 vaccination in Uganda has been majorly in government healthcare facilities. This accessibility to vaccination services could have led to higher uptake among HCWs in these facilities. While this study was conducted among adults, similar studies among children have reported that accessibility to vaccine services played a key role in completion of vaccination schedules [43]. The participation in COVID-19 vaccine related activities was associated with increased uptake of the vaccines. Participating in such activities builds knowledge and awareness of the vaccines, their benefits, and safety which further builds vaccine confidence and trust. HCWs who have been vaccinated are more likely to encourage their clients/patients to get vaccinated than the hesitant workers thus improving uptake by the public. However, no studies have been done in this area hence a need for further research.

The study further explored the relationship between previous testing for SARS-COV-2 and vaccine uptake among HCWs. We found out that HCWs who had ever tested for SARS-COV-2 were more likely to take the vaccines than those who had never tested. Similar findings were reported by Mazin *et al*. 2021 [44]. The likelihood of testing for the corona virus infection is probably higher among those with higher risk perception such as HCWs. Contrary to Mazin *et al*. 2021 [44] who reported that participants who had a negative test result were more likely to be vaccinated, our study did not observe any difference in vaccine uptake among those with either positive or negative test result. Testing positive was possibly taken as natural immunization, therefore, the HCWs didn’t see the need to be vaccinated. More to this, there has been theoretical belief of immune enhancement of disease implying that those that tested positive could have feared to take the vaccine.

## Conclusion and recommendations

The study reported a high level of uptake of vaccines among HCWs within Entebbe municipality due to accessibility to vaccines. However, there remains a significant proportion of HCWs who are hesitant to take vaccination therefore, further studies are needed to understand and better address the reasons for the vaccine hesitancy. Since COVID-19 vaccine uptake was associated with accessibility and participation in COVID-19 vaccination services, the government and development partners should ensure that vaccination services are equitably distributed to ensure access to all those in need. The earlier involvement of private health facilities in the COVID-19 vaccination campaign could have improved vaccine uptake not only amongst HCWs but also in the public. Further studies are recommended to explore drivers of vaccine hesitancy among HCWs.

## Strengths and limitations

This study is among the first few to explore actual vaccine uptake among health care workers in SSA and collected data from front line primary health care workers during the peak of the Delta variant of SARS-CoV-2 wave, therefore all barriers or motivators for vaccine uptake would be most expressed during this period. Thus, our findings are a true reflection of vaccine uptake during an outbreak. The study was carried out during the country wide lock down, which would have hindered participation, but since HCWs were allowed to work, enrolment and participation were not affected. Approximately 99% of the calculated sample size responded. Also, for participants with access to smart phones or internet connected computers, the online version of the questionnaire was sent to them which they completed and sent back. This hybrid approach to data collection increased participation in the study. However, having been vaccinated was reported, and the study team could not verify the vaccination status.

## Study implication

This study reveals that intention to vaccinate does not translate into actual vaccine uptake, therefore inherent vaccine hesitancy prevails even when vaccines are accessible, available and among individuals with adequate knowledge of vaccines. Exploring vaccine hesitancy should therefore be an ongoing activity especially with new vaccines, in different contexts, and among different populations.

## Supporting information

S1 Data (XLSX)
